# Memory, Attention, and Concentration Dysfunction Post-COVID-19 Among College Students in Saudi Arabia: A Case-Control Study

**DOI:** 10.7759/cureus.36419

**Published:** 2023-03-20

**Authors:** Zainah A Al-Qahtani, Imtinan Al Jabbar, Wajd Alhadi, Seham A Alahmari, Rawan M Alqahtani, Bayan M Alnujaymi, Reem A Al-Qahtani

**Affiliations:** 1 Department of Internal Medicine, King Khalid University, Abha, SAU; 2 College of Medicine, King Khalid University, Abha, SAU

**Keywords:** case control studies, post-covid sequelae, the kingdom of saudi arabia, impact on academic performance, long covid, cognitive deficit, covid 19

## Abstract

Introduction

Multiple studies have demonstrated the multi-systemic involvement of COVID-19, and among all of these systems, there is mounting evidence that COVID-19 is linked to neurocognitive impairment, particularly when neurological symptoms are present. Our aim is to study the concept of cognitive dysfunction post-COVID-19 among college students in Saudi Arabia and its potential effect on their academic performance.

Methods

A population-based, observational case-control study was conducted across the Kingdom of Saudi Arabia, from May 2022 to September 2022. A total of 2,150 eligible students have completed the study questionnaire. An exact 776 (36.1%) of them had COVID-19 infection (group 1), while 1,374 (63.9%) students had not (group 2). The sample population was college-enrolled students from 18 to 28 years old, with a mean age of 21.3 for group 1 and 20.8 for group 2. Both groups were handed the same data collection tool to establish whether the COVID-19 survivors had cognitive deficits more than the control group.

Results

There was no significant difference between the two groups regarding their bio-demographic data, study methods, or vaccination rate. However, both Neurological Fatigue and Big Five Inventory score were significantly higher among infected students, comparable to non-infected students. A negative relation was found between the infected students’ neurological fatigue (rho=-0.14), cognitive failure (rho=-0.10), and depression and anxiety scale with their GPA (rho=0.03). Contrarily, infected students showed a positive relationship between their GPA and the Big Five Inventory (rho=0.13) and Short Grit Scale (rho=0.14). Also, there was a significant inverse relation between students’ apathy motivation with their Big Five Inventory. Likewise, there was an inverse relation between their neurological fatigue, cognitive failure, and apathy motivation with their Short Grit Scale.

Conclusion

We demonstrated that college students who have survived COVID-19 infection mostly complain of cognitive impairment, even though most of them have no comorbidities or psychological disorders.

## Introduction

COVID-19 is a viral infection that had spread throughout the world and has since become a global public health concern. According to WHO, 759,408,703 cases had been confirmed, including 6,866,434 deaths [1]. The clinical presentation mainly affects the respiratory tract, but current studies demonstrate the presence of multi-systemic involvement [2], the systemic effects of this viral disease are not only confined to the period of overt illness but also in its recovery period; this is what is recognized as the post-COVID-19 syndrome [3]. Some presentations have been found to involve the neurological system [2]. The lasting neurological symptoms in question are ones afflicting the most sophisticated levels of mental function which are the cognitive ones. Cognitive functions are a performance of higher mental operations that work to aid humans to gather and process information from external stimuli [4].

Some of the most important and noticeable cognitive functions that have been linked to various degrees of decline in the recovery period of COVID-19 are memory, attention and concentration [5]. It has been established in different publications that patients who had mild symptoms have shown memory related issues sustained post-COVID-19 recovery when followed up after eight months [6].

The aim of this study is to shed light on the concept of cognitive dysfunction post-COVID-19 recovery among college students as it is under-researched topic in this context, particularly in Saudi Arabia, and the subjects are constantly in an academic environment and are aware and able to report their symptoms.

## Materials and methods

A population-based, observational case-control study was conducted across the Kingdom of Saudi Arabia from May 2022 to September 2022. The study compared the cognitive function and academic performance of college students who had COVID-19 to those who have not encountered the infection in different areas of Saudi Arabia. Both groups were handed the same data collection tool to establish whether the COVID-19 survivors have cognitive deficits more than the control group.

Study population

Our target population was college students who have encountered COVID-19 as well as their age-matched non-infected student counterparts in 42 colleges across different areas of Saudi Arabia.

Inclusion and exclusion criteria 

College students of both genders aged 18 years old or more from different areas of Saudi Arabia were included in this study, then classified according to whether they were infected or not into two groups. Participants who were admitted to the hospital were excluded, to avoid any potential bias as recent literature has shown that hospital stay can cause some sort of cognitive dysfunction [7]. Also, participants who had mental illnesses did not complete the entire questionnaire or did not consent to participate were excluded from the study. 

Data collection tool and procedure

The data collection tool was composed of ten sections. The first section included biographical information such as age, gender, region, specialty as well as the year of study. The second section included questions to categorize the participants into the two required groups, as follows: “Have you been infected with COVID-19?”, “When were you infected?”, “What symptoms have you experienced?”, “Where did you spend the period of the infection?”, “Have any of the symptoms continued for more than 4 weeks after you first had COVID-19, that are not explained by something else?”. The third section included questions about academic performance before and after the COVID-19 infection. The fourth section included questions about the COVID-19 vaccine such as “Have you been vaccinated against COVID-19?”, “Date of the most recent vaccination?”, “Which type of vaccination?”, “How many doses did you get?”. The fifth section is to assess the relationship between sleep and fatigue by using the Neurological Fatigue Index (NFI), this questionnaire has been used in a previous study for fatigue evaluation in multiple sclerosis patients [8]. The sixth section is to assess the forgetfulness and simple mistakes that happen during day-to-day activities in the past two weeks by using the Cognitive Failures Questionnaire (CFQ) [9]. The seventh section is to assess emotional, behavioral, and social apathy by using the Apathy-Motivation index (AMI) [10]. The eighth and ninth sections were meant to assess the personality by using the Big Five Inventory (BFI-S) [11], and the Short Grit Scale (GRIT-S) [12]. Both indices are used to measure the conscientiousness personality trait and persistence as well as dedication to attain a desired goal, but with different questions so that the most accurate results are achieved. The tenth section included the Hospital Anxiety and Depression Scale (HADS) [13], which has been designed to help us to know how the participant feels.

The data collection was done via well-trained data collectors numbered approximately 100 across the Kingdom of Saudi Arabia from different colleges and specialties. Each collector was asked to collect 30 responses, of which 20 responses were from control participants (group 2) while the remaining 10 responses were from case participants (group 1). The tasks given were accomplished within a time window from May 9 to September 7, 2022. The collectors were employed to ensure the completion of the questionnaires by the participants in a face-to-face setting which adds to the integrity of the study. Upon the completion of the data collection, the collectors received a certificate for their efforts and were awarded the points they deserved by the Saudi Commission for Health Specialties (SCHS). All questionnaires used in this study were previously validated as they were taken from existing publications after obtaining permission.

In this study, various efforts have been made to prevent any potential sources of bias. Firstly, avoid the submission of duplicate or fake answers by making the data collection tool accessible only once per person. Secondly, the data collectors obtained the required information by handing the data collection tool to the participants in a face-to-face setting, thus maintaining the integrity of the sample’s answers. Thirdly, in this case-control study, both groups of the sample population were randomly selected from different colleges and specialties across the Kingdom of Saudi Arabia so as to avoid selection bias. Lastly, the study was conducted in all regions of Saudi Arabia, therefore achieving the most diverse and accurate results irrespective of geographical standing.

All information was kept private, and consent from the participants was obtained before participating in the study. Ethical approval of the study was obtained from the Research Ethics Committee at King Khalid University (Issued approval: ECM#2022-1201).

Data management and statistical analysis

After data were extracted, it was revised, coded, and fed to statistical software IBM SPSS version 22 (SPSS, Inc. Chicago, IL). All statistical analysis was done using two-tailed tests. P value less than 0.05 was statistically significant. For different scales, the overall score was obtained by summing up all item scores (after reversing negative statement scores if there was) reference to scales given scoring system. Descriptive analysis based on frequency and percent distribution was done for all variables including participants' personal data, smoking habits, and co-morbidities among study groups. Also, students' academic performance data and COVID-19-related data were compared among study groups. Cross-tabulation was used for all these relations using Persons’ chi-square test and exact probability test for small frequency distributions. Memory, cognitive, and psychological dysfunction scores were presented as mean with standard deviation using independent samples t-test for comparison significance. Correlation analysis was used to assess the Correlation between students' GPA and cognitive, memory, and psychological dysfunction among study groups and also the correlation between students' cognitive, memory, and psychological dysfunction among study groups.

## Results

A total of 2150 eligible students completed the study questionnaire. Exact of 776 (36.1%) had COVID-19 infection (group 1) while 1,374 (63.9%) had not (group 2). There was no significant difference between the two study groups regarding their age (21.3 vs 20.8 mean age), gender distribution, body mass index, smoking status, chronic health problems according to Table 1. 

**Table 1:** Bio-demographic data of study college students' groups, Saudi Arabia P: Pearson X2 test                                   $: Exact probability test

Bio-demographic data	Group				p-value
	Covid-19 infected		Covid-19 non-infected		
	No	%	No	%	
Age in years					.957
18-20	122	15.7%	213	15.5%	
21-22	469	60.4%	828	60.3%	
23-25	185	23.8%	333	24.2%	
Gender					.166
Male	190	24.5%	374	27.2%	
Female	586	75.5%	1000	72.8%	
Body mass index					.552
Underweight	103	13.3%	169	12.3%	
Normal weight	457	58.9%	802	58.4%	
Overweight	141	18.2%	244	17.8%	
Obese	75	9.7%	159	11.6%	
Smoking					.821
Yes	65	8.4%	119	8.7%	
No	711	91.6%	1255	91.3%	
Chronic diseases					.924^$^
DM	7	.9%	15	1.1%	
HTN	4	.5%	8	.6%	
Asthma	44	5.7%	79	5.7%	
Others	16	2.1%	36	2.6%	
None	705	90.9%	1236	90.0%	

Moreover, Table 2 shows no significant difference between the study groups regarding the study field, study method (physical /* virtual), or even in the dominant brain side. Also, GPA was nearly uniform distributed among the two study groups especially for high grades (P>0.05 for all). 

**Table 2:** Study data of college students' groups, Saudi Arabia P: Pearson X2 test                                   $: Exact probability test

Study data	Group				p-value
	Covid-19 infected		Covid-19 non-infected		
	No	%	No	%	
Study field					.978
Health related field	287	37.0%	509	37.0%	
Non-health related field	489	63.0%	865	63.0%	
Study method					.754^$^
Physical	472	60.8%	838	61.0%	
Virtual	12	1.5%	16	1.2%	
Both of them	292	37.6%	520	37.8%	
Are you Rt / Lt handed					.579
Right handed	683	88.0%	1188	86.5%	
Left handed	69	8.9%	136	9.9%	
Both	24	3.1%	50	3.6%	
GPA					.901
A+ / A	335	43.2%	579	42.1%	
B+ / B	325	41.9%	577	42.0%	
C+ / C	93	12.0%	179	13.0%	
D+ / D	23	3.0%	39	2.8%	

Table 3 demonstrates the COVID-19 vaccination data among college students' groups, an exact of 99.6% of students who had COVID-19 infection were vaccinated versus 99.1% of others with no statistical significance (P=0.147). Additionally, no significant difference was reported for duration since vaccination nor for the type of received vaccines. 

**Table 3:** Covid-19 vaccination data among college students' groups, Saudi Arabia P: Pearson X2 test

Covid-19 vaccination	Group				p-value
	Covid-19 infected		Covid-19 non-infected		
	No	%	No	%	
Had covid-19 vaccine?					.147
Yes	773	99.6%	1361	99.1%	
No	3	.4%	13	.9%	
Year of last dose					.112
2020	29	3.8%	78	5.7%	
2021	265	34.3%	441	32.4%	
2022	479	62.0%	842	61.9%	
Type of the vaccine					.096
Pfizer	712	92.1%	1242	91.3%	
Moderna	123	15.9%	219	16.1%	
AstraZeneca	227	29.4%	354	26.0%	

**Figure 1:**
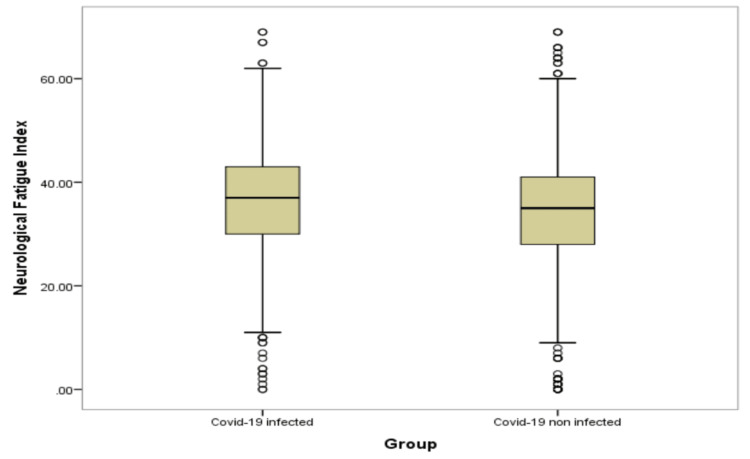
Box-plot for neurological fatigue among students' groups

**Figure 2:**
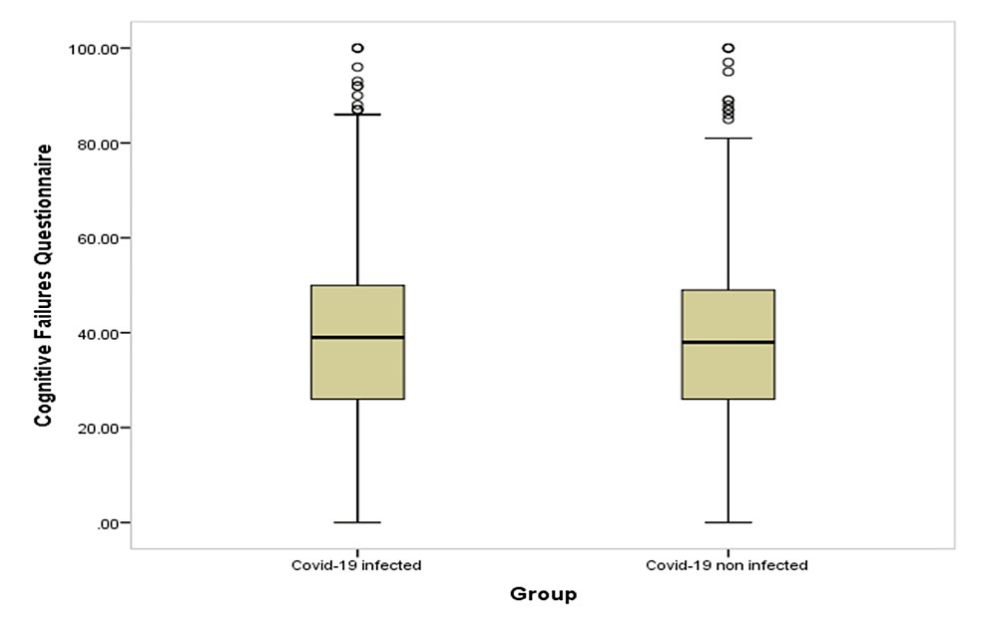
Box-plot for cognitive failure among students' groups

**Figure 3:**
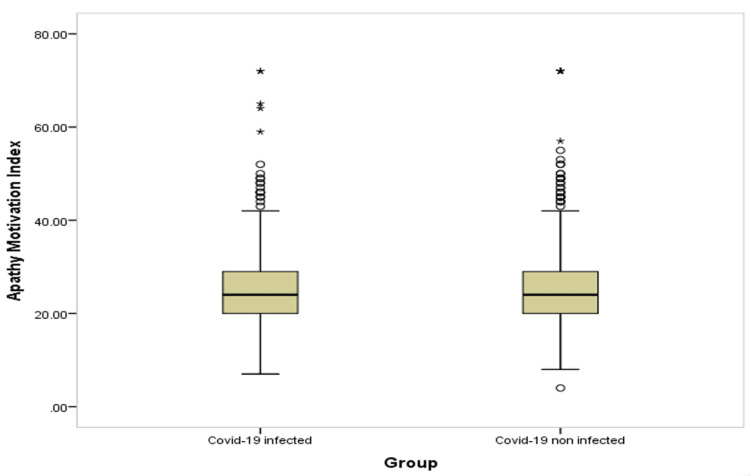
Box-plot for apathy motivation function among students' groups

**Figure 4:**
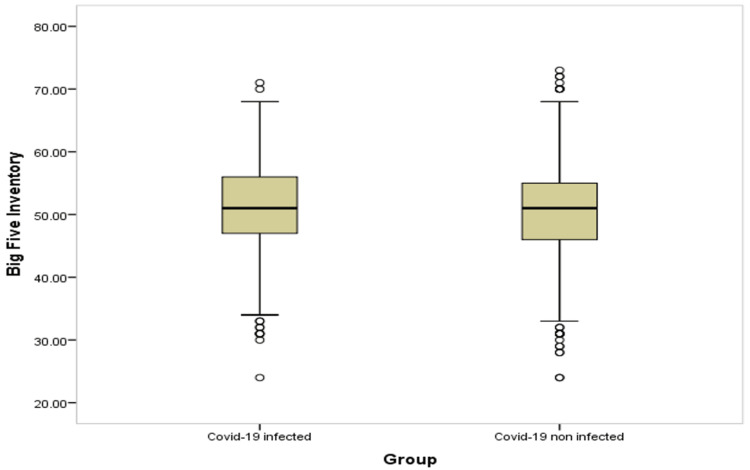
Box-plot for big five inventory among students' groups

**Figure 5:**
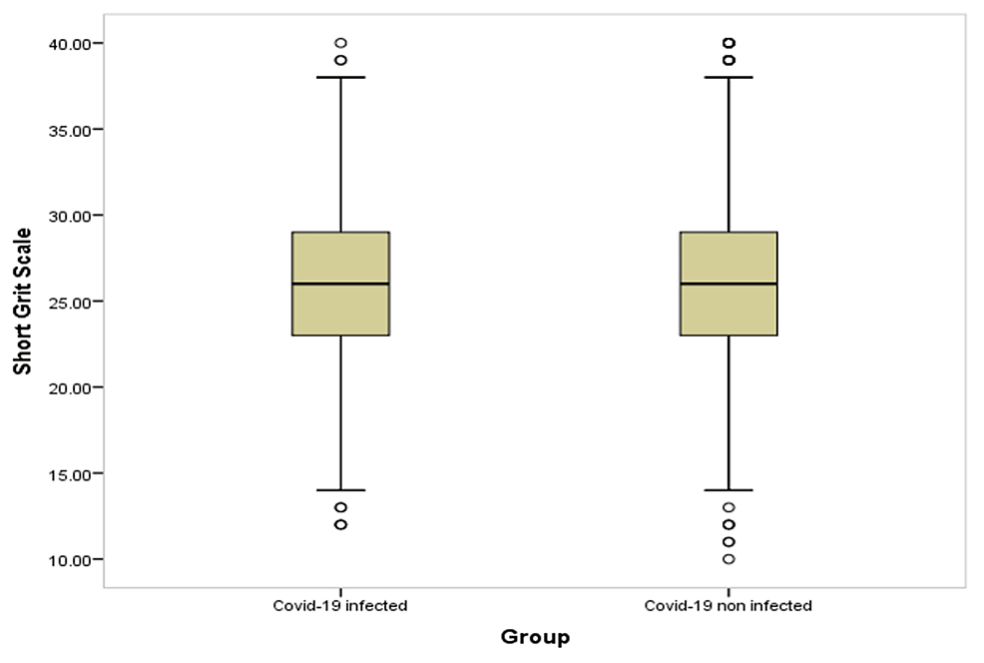
Box-plot for short grit scale among students' groups

**Figure 6:**
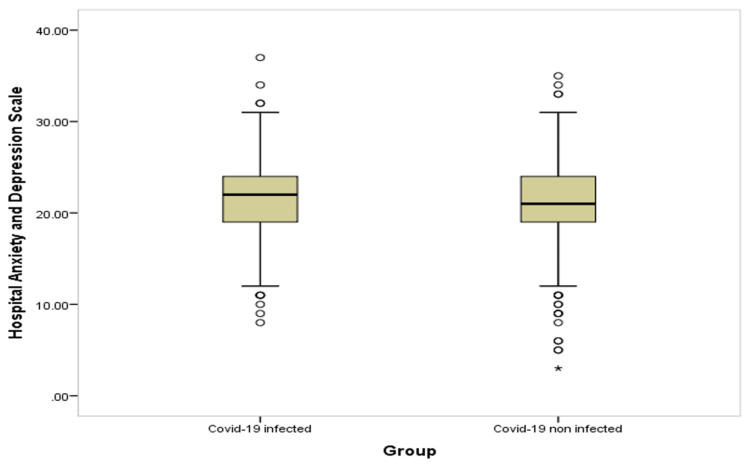
Box-plot for anxiety and depression among students' groups

Table 4 and Figures 1-6 show significant difference between the two study groups at neurological fatigue with mean score of (36.2 11.0) for infected students compared to (34.5 11.3) for non-infected students (P=0.001). Also, Big Five Inventory score was higher among infected students than among non-infected group (51.2 6.8 vs. 50.6 6.8, respectively; P=0.033). Other cognitive functions and psychological functions were nearly equal among the two study groups with no significant difference (P>0.05 for all).

**Table 4:** Memory, attention and concentration dysfunction among college students' groups, Saudi Arabia P: Independent samples t-test * P < 0.05 (significant)

Neurological, cognitive and psychological assessment	Group				p-value
	Covid-19 infected		Covid-19 non-infected		
	Mean	SD	Mean	SD	
Neurological Fatigue Index	36.2	11.0	34.5	11.3	.001*
Cognitive Failures Questionnaire	38.6	18.4	37.8	17.2	.319
Apathy Motivation Index	25.3	7.7	25.4	8.0	.758
Big Five Inventory	51.2	6.8	50.6	6.8	.033*
Short Grit Scale	26.0	4.7	26.1	4.8	.605
Hospital Anxiety and Depression Scale	21.4	3.8	21.3	3.8	.749

Table 5 represents the correlation between student's GPA and neuro-cognitive, memory and psychological dysfunction among study groups. Among students with COVID-19 infection, there was a significant inverse relation between students' neurological fatigue (rho=-0.14), cognitive failure (rho=-0.10), and depression and anxiety with their GPA (rho=0.03). The positive relations of GPA were with Big Five Inventory (rho=0.13) and with Short Grit Scale (rho=014). As for the other students, neurological fatigue the only function that negatively affected GPA (rho=-0.08) while Big Five Inventory (rho=0.07) and with Short Grit Scale (rho=0.13) showed positive relations.

**Table 5:** Correlation between students’ GPA and neurocognitive, memory, and psychological dysfunction among study groups rho: Spearman correlation coefficient * P < 0.05 (significant)

Group	Scale	GPA	
		rho	P-value
Covid-19 infected	Neurological Fatigue Index	-.14	.001*
	Cognitive Failures Questionnaire	-.10	.007*
	Apathy Motivation Index	-.06	.075
	Big Five Inventory	.13	.001*
	Short Grit Scale	.14	.001*
	Hospital Anxiety and Depression Scale	-.03	.337
Covid-19 non-infected	Neurological Fatigue Index	-.08	.002*
	Cognitive Failures Questionnaire	-.04	.100
	Apathy Motivation Index	-.05	.053
	Big Five Inventory	.07	.007*
	Short Grit Scale	.13	.001*
	Hospital Anxiety and Depression Scale	-.04	.130

As for Table 6, it explains the correlation between student's neuro-cognitive, memory, and psychological dysfunction among study groups. Both groups had a significant inverse relation between students’ apathy motivation with their big five inventory. Likewise, there was inverse relation between their neurological fatigue, cognitive failure and apathy motivation with their Short Grit Scale.

**Table 6:** Correlation between students’ neurocognitive, memory, and psychological dysfunction among study groups r: Pearson correlation coefficient * P < 0.05 (significant)

Correlations	Scale	Big Five Inventory		Short Grit Scale	
		r	P	r	P
Covid-19 infected	Neurological Fatigue Index	.09	.012*	-.27	.001*
	Cognitive Failures Questionnaire	.07	.051	-.39	.001*
	Apathy Motivation Index	-.45	.001*	-.24	.001*
	Hospital Anxiety and Depression Scale	.02	.496	.24	.001*
Covid-19 non-infected	Neurological Fatigue Index	.12	.001*	-.24	.001*
	Cognitive Failures Questionnaire	.01	.832	-.39	.001*
	Apathy Motivation Index	-.38	.001*	-.25	.001*
	Hospital Anxiety and Depression Scale	.09	.001*	.21	.001*

**Figure 7:**
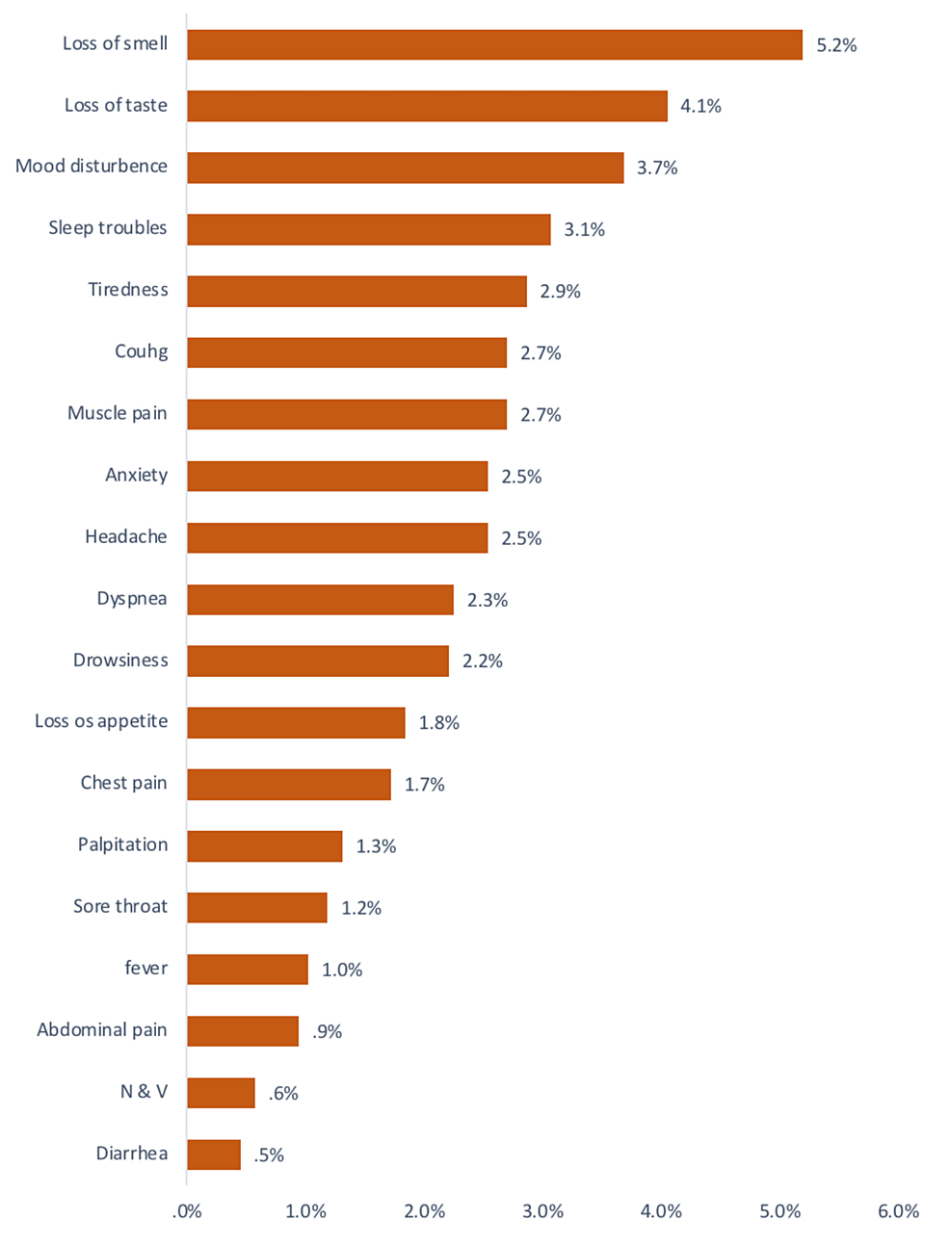
Symptoms continued after COVID-19 infection

**Figure 8:**
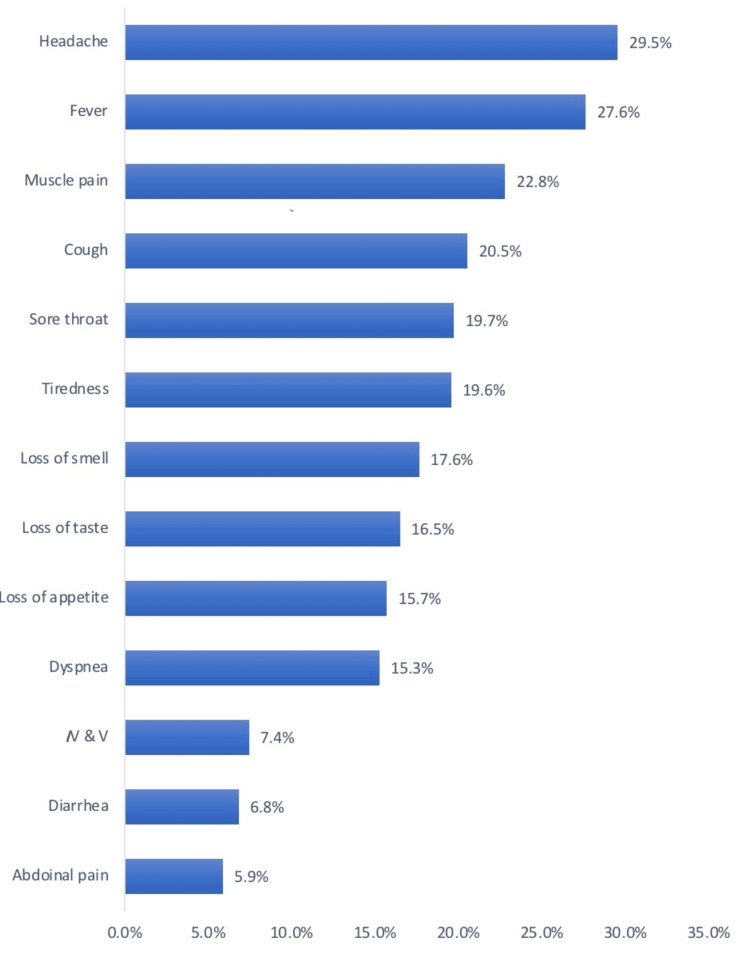
Symptoms during COVID-19 infection

Among the symptoms that continued after the infection, the cognitive ones were present as demonstrated in Figure 7. The symptoms that had been experienced during the infection are shown in Figure 8.

## Discussion

Post-COVID-19 syndrome is defined as persistent clinical signs and symptoms that emerge during or after infection with COVID-19 [3,14]. These symptoms continue for more than 12 weeks [14]. Although variable symptoms were reported, cognitive impairment was one of the most concerning persistent symptoms experienced by many individuals after encountering the infection [3,14]. This forms an obstacle in performing day-to-day activities, especially for college students who need the best cognitive potential.

To our knowledge, there is no quantitative study in the recent literature that has established the effect of COVID-19 on the cognitive status and academic performance of college students. In this study, we measured the cognitive abilities of college students who survived COVID-19 through multiple questionnaire-based measures (NFI, CFQ, AMI, BFI-S, GRIT-S, HADS) [8-13] and compared them to age-matched controls, with considerations for the gender distribution, body mass index, smoking status, chronic health problems, psychological disorders, study field, study method(physical/*virtual) and vaccination status of both study groups. There was no significant difference in these parameters as demonstrated in Tables 1-3.

The most persistent cognitive symptoms among COVID-19 survivors in our study were the following: mood disturbance in 3.7%, sleep troubles in 3.1%, tiredness in 2.9%, anxiety in 2.5%, headache in 2.5%, drowsiness in 2.2%, along with other general symptoms as shown in Figure 7. These findings are concordant with the publication of Visco et al. [15] which has shown that COVID-19 survivors frequently exhibit neurological and psychiatric sequelae.

Neurological fatigue was significantly higher among infected students, comparable to non-infected students. Similarly, the Big Five Inventory score which describes an individual’s consistent efforts and dedication to attain a particular goal was higher among infected students (Table 4). Apart from that, no significant difference was noted between the two groups. A negative relation was found between the infected students’ neurological fatigue (rho=-0.14), cognitive failure (rho=-0.10), and depression and anxiety scale with their GPA (rho=0.03). Contrarily, infected students showed a positive relationship between their GPA and the Big Five Inventory (rho=0.13) and Short Grit Scale (rho=0.14). As for the non-infected students, neurological fatigue was the only factor that affects their GPA negatively (Table 5). Among both groups, there was a significant inverse relation between students’ apathy motivation with their Big Five Inventory. Likewise, there was an inverse relation between their neurological fatigue, cognitive failure, and apathy motivation with their Short Grit Scale (Table 6).

Our findings are consistent with those of other studies, in which cognitive dysfunction including impaired concentration and poor memory is demonstrated as one of the concerning complications in 18%-50% of the patient after encountering the infection [16-18]. Patients who had mild symptoms still show memory-related issues when followed up after eight months [6].

However, our study is not without its limitations. Firstly, the lack of a proper neurological clinical examination and vigilance test conduction may have influenced the outcomes of the study, where a further increase in cognitive dysfunction among the infected students could have been detected as shown in the publication of Zhao et al. [19]. Secondly, participants were only asked whether they have been infected or not without conducting any further confirmatory tests, under the assumption that individuals had received the diagnosis from a PCR test clinically. This limited action was due to a lack of funding received for the research. Lastly, a potential improvement in the cognitive performance of the sample might be shown if further follow-up was conducted.

## Conclusions

We demonstrated that college students who have survived COVID-19 infection mostly complain of cognitive impairment, even though most of them have no comorbidities or psychological disorders. However, the good news is that their academic performance was not remarkably affected despite their impaired cognitive function. Our recommendation for future articles is to assess the improvement of cognitive function among COVID-19 survivors.
